# Impact of migrating from paired to sequential stimulation on speech recognition in cochlear implant users

**DOI:** 10.1016/j.bjorl.2026.101910

**Published:** 2026-09-22

**Authors:** Aline Faria de Sousa, Aline Santos, Lucas Bevilacqua Alves da Costa, Orozimbo Alves Costa, Ana Claudia Martinho de Carvalho

**Affiliations:** a Alfa Instituto de Comunicação e Audição, São Paulo, SP, Brazil; b Pontifícia Universidade Católica de São Paulo (PUC), Faculdade de Medicina, Sorocaba, SP, Brazil; c Universidade de São Paulo (USP), Faculdade de Odontologia (FOB), Departamento de Fonoaudiologia, Bauru, SP, Brazil; d Advanced Bionics LLC, Valencia, California, United States

**Keywords:** Cochlear implants, Auditory perception, Hearing loss, Speech perception

## Abstract

**Objective:**

For many years, the paired stimulation strategy (P) was the standard configuration recommended by Advanced Bionics for cochlear implant users. However, recent evidence has demonstrated advantages of the sequential strategy (S), leading the company to adopt it as the new standard. Despite this change, many users continue to rely on the P strategy. In this context, the present study aimed to determine whether the S strategy truly provides advantages for users who have been using the P strategy for at least three months. Notably, no studies on this topic have been conducted in Portuguese or other Latin-based languages.

**Methods:**

Nine cochlear implant users participated in the study, totalling eleven ears analyzed. All participants initially used maps programmed with the P strategy, with routine clinical follow-up. These maps were configured based on each user's measured comfort level (M). After reprogramming to the S strategy, the maximum stimulation levels (M) were readjusted according to individual comfort reports. Detection thresholds (T) were set at 10% of the M value for both the original and the updated maps. Each ear underwent a sentence recognition test using the P strategy and, subsequently, the S strategy, allowing for direct comparison of performance. For this comparison, the paired Student's *t-test* was used, the level of significance adopted was 5% (p < 0.05).

**Results:**

All participants demonstrated improved performance with the S strategy. The difference between strategies was statistically significant, with an average increase of 23% in sentence recognition following the transition to sequential stimulation.

**Conclusion:**

The results indicate that the sequential strategy provides significantly better performance than the paired strategy.

**Level of evidence:**

2.

## Highlights

• Sequential stimulation improved speech recognition in CI users.
• Optima-S outperformed Optima-P in within-subject analysis.
• First Portuguese study comparing Optima-P and Optima-S.

## Introduction

The Cochlear Implant (CI) is a technology developed for individuals with severe to profound sensorineural hearing loss who have not obtained satisfactory benefits from the use of conventional hearing aids. Over the years, this technology has evolved significantly, providing better speech intelligibility and a higher quality of life for its users.

The companies responsible for developing CI have sought to constantly improve clinical results by investing in research focused mainly on the external component of the device ‒ the speech processor. It is in this component that the acoustic signal is encoded and transformed into electrical impulses, which are then delivered to the implant electrodes. With the advent of Continuous Interleaved Sampling (CIS) signal processing, it has been possible to achieve better audiological performance among CI users.^1^^,^^2^ This strategy has reduced interference between stimulation channels and allowed for more efficient delivery of the speech envelope. Based on this advance, new processing algorithms were developed with the aim of improving the representation of the fine temporal aspects of the acoustic signal, further contributing to the improvement of speech perception.

Advanced Bionics (AB) currently offers six sound processing strategies in its CI mapping software. Among these, the most recent and widely used are the HiRes Optima-P and HiRes Optima-S strategies. The paired (P) mode stimulates two channels simultaneously, and in the sequential (S) mode, each channel is stimulated successively. Although the HiRes-P modality has been the most widely used strategy for the initial activation of new users for many years, technological advances and the latest clinical evidence highlight superior speech recognition results when using the S strategy compared to the benefits obtained with the P strategy.^3^

Comparative studies have shown significant advantages of sequential stimulation (S) strategies over paired (P) strategies, especially in speech recognition tasks in quiet and competitive noise environments. Buechner et al. (2005)^4^ observed better performance in word and sentence recognition in noise with the use of the sequential strategy compared to the paired strategy. Similarly, Reynolds and Gifford (2019) identified greater subjective preference and better speech perception scores among users undergoing sequential stimulation.^5^

Given these results, it is questioned whether users who have been using the P strategy for some time could benefit from switching to the S strategy, especially those whose hearing performance is not fully satisfactory. The literature suggests that, even in experienced users, strategic adjustments can promote significant perceptual improvements, increasing the potential for intelligibility and hearing comfort. However, the absence of studies conducted in Portuguese- and Spanish-speaking populations is an important limitation in extrapolating these results to Latin American contexts. There is, therefore, a need for local clinical research that systematically evaluates the comparative effects of the Optima-S and Optima-P strategies, contributing to a culturally contextualized clinical practice.

Given the above, the present study aimed to compare speech recognition between the HiRES-Optima P and HiRES-Optima S strategies in adolescents and adults using CI.

## Methods

### Participants

This prospective pre–post comparative study was conducted in 2025 at the Prevent Senior.

The sample was convenience-based, consisting of cochlear implant users followed up at a specialized hearing rehabilitation service. Participants were included consecutively as they attended the service during the data collection period, provided they met the eligibility criteria described below.

The inclusion criteria were: being at least 14-years of age; having been initially activated with the P strategy and remaining in use of this strategy since device activation; having at least three months of cochlear implant use; and being available to participate in scheduled evaluations.

The study included nine cochlear implant users, totaling eleven ears evaluated. Six participants used the device unilaterally. Among the four bilateral users, two had both ears evaluated separately. For the other two, only one ear was included in the analysis, as the contralateral ear map showed additional changes beyond the measurement of comfort level and strategy change, which made it impossible to compare the strategies. All tests were performed unilaterally, considering each ear as an independent unit for statistical analysis.

The tests were conducted in an acoustic booth, with the cochlear implant user positioned one metre from the loudspeaker at 0° azimuth in both the horizontal and vertical planes. Stimuli were presented using a Madsen Astera 2 audiometer (Otometrics). For participants evaluated without noise ‒ who comprised the majority of the sample ‒ the speech stimulus was presented in a free field at 60 dB SPL. Most participants were assessed in silence because their initial performance did not exceed 80%. When performance exceeded this threshold, and according to the service protocol, the assessment was conducted in the presence of competitive noise, as this condition is more challenging and better reflects everyday communication demands.

All participants took the Portuguese Sentence Lists (LSP) test.^6^ For one participant, the slow version of the test (LSP-L)^7^ was applied due to difficulties observed with the typical version of the LSP. For another participant, the test was performed at a signal-to-noise ratio of +5 dB, with the speech signal presented at 65 dB NPS, due to better performance in the silent condition.^8^

### Collection procedures

All participants used maps configured in the P strategy, with updated clinical follow-up. The maps in use were based on the measurement of each user's comfort level (M).

After switching to the new stimulation strategy (S strategy), the maximum stimulation levels (M) were adjusted according to the comfort level reported by each participant.

Detection thresholds (T) were set at 10% of the M value for both the map in use and the map resulting from the strategy change.

Each ear underwent two speech recognition assessments, performed at the following times: 1) Using strategy P, prior to switching to strategy S, using the map in use (baseline); 2) At the time of the strategy change or up to one month after using strategy S.

### Test procedures

The assessments were performed in a treated acoustic booth, in the unilateral cochlear implant condition, with the stimulus presented at 0° azimuth and a fixed intensity of 60 dB NPS. The lists were presented in recorded format, ensuring reproducibility between the different assessment times. For the individual who performed the test in noise, speech was presented at 65 dB NPS and noise at 60 dB NPS; The list used in each session was randomly selected. Performance analysis considered the score for words correctly recognized in the sentences.

### Ethical aspects

All participants and the guardians of underage participants signed the Free and Informed Consent Form (FICF) before data collection, authorizing the use of their data for scientific research purposes, as approved by the institution's Research Ethics Committee under opinion number 7.451.722.

### Data analysis

The statistical analysis considered each ear evaluated as a unit of analysis. Speech recognition test performance data were compared intra-individually, considering the moments of evaluation before and after the change in processing strategy.

For this comparison, the paired Student's *t-test* was used, after verifying the assumption of normality using the Shapiro-Wilk test. The level of significance adopted was 5% (p < 0.05).

In addition to *the t-value* and significance level, the effect size was analyzed using Hedges' g, a measure derived from Cohen's *d* that includes correction for bias in small samples. Hedges' *g*-values were interpreted according to the criteria proposed in the literature, with effect sizes corresponding to *g* = 0.25, *g* = 0.55, and *g* = 0.95 being considered small, medium, and large, respectively.^9^ The 95% Confidence Intervals (95% CI) for the mean difference between conditions and for the effect size were also estimated in order to assess the accuracy of the estimates.

## Results

Table 1 presents the demographic data of the sample, such as age, duration of use of the P and S strategy, etiology of hearing loss, type of deafness, ear evaluated and test applied.

**Table 1:** Demographic data of the sample.

ID	Gender	Age	Etiology of hearing loss	Deafness	Ear	Time using P strategy (months)	Internal Component	Processor	Test Used	Time using strategy S	P % correct	S % correct
1	F	34	Idiopathic	Pre-lingual	Right	10	Hires Ultra 3D Slim J	M90	LSP	1 day	25.74	33.3
2	M	27	Idiopathic	Postlingual	Right	7	Hires Ultra Slim J	M30	LSP	1 month	19.2	63.8
3	M	46	Ototoxicity	Lingual powder	Right	26	Hires Ultra 3D Slim J	M90	LSP	1 day	72.54	100
					Left	3	Hires Ultra 3D Slim J	Q90	LSP	1 day	78	91.02
4	F	48	Idiopathic	Postlingual	Left	51	Hires Ultra 3D Slim J	M90	LSP	1 day	46.8	65.52
5	F	72	Idiopathic	Postlingual	Right	23	Hires Ultra Slim J	Q70	LSP-L	1 day	4.8	30.16
6	F	66	Idiopathic	Post-lingual	Left	30	Hires Ultra Slim J	Q70	LSP	1 month	58.83	96.47
7	F	43	Idiopathic	Pre-lingual	Left	28	Hires Ultra 3D Slim J	Q90	LSP	1 month	73.87	76.47
					Right	17	Hires Ultra 3D Slim J	Q90	LSP	1 month	50.59	77.91
8	F	41	Idiopathic	Postlingual	Right	8	Hires Ultra 3D Slim J	Q90	LSP	1 month	76.09	79.92
9	M	16	Idiopathic	Postlingual	Right	4	Hires Ultra 3D Slim J	M30	LSP-S-R+5	1 day	68.89	84.71

Table 2 shows the descriptive statistics of performance in speech recognition tests, considering the P (pre-change) and S (post-change) strategies. The values are expressed as a percentage (%) and include the mean, first quartile (Q1), median, and third quartile (Q3).

**Table 2:** Performance in speech recognition tests according to the strategy used.

Strategy	Mean (%)	Q1 (%)	Median (%)	Q3 (%)
P (pre-change)	52.30	36.27	58.83	73.21
S (post-change)	72.66	64.66	77.91	87.87

The paired *t*-test was conducted to compare speech recognition performance between the P (pre-change) and S (post-change) strategies. The assumptions of data normality were met, according to the Shapiro-Wilk test (p > 0.05) for both conditions. The paired *t*-test analysis and effect size can be seen in Table 3 (Fig. 1).

**Table 3:** Comparison of performance between P and S strategies.

Comparison	Test	*t* (gl)	p	Mean difference (%)	95% CI of difference	Hedges’ *g*	95% CI of Hedges’ *g*
S vs. P	Paired Student's *t*-test	4.98 (10)	<0.001	20.36	11.25–29.47	1.38	0.56–2.18

df, Degrees of freedom; 95% CI, 95% Confidence Interval.

**Fig. 1:**
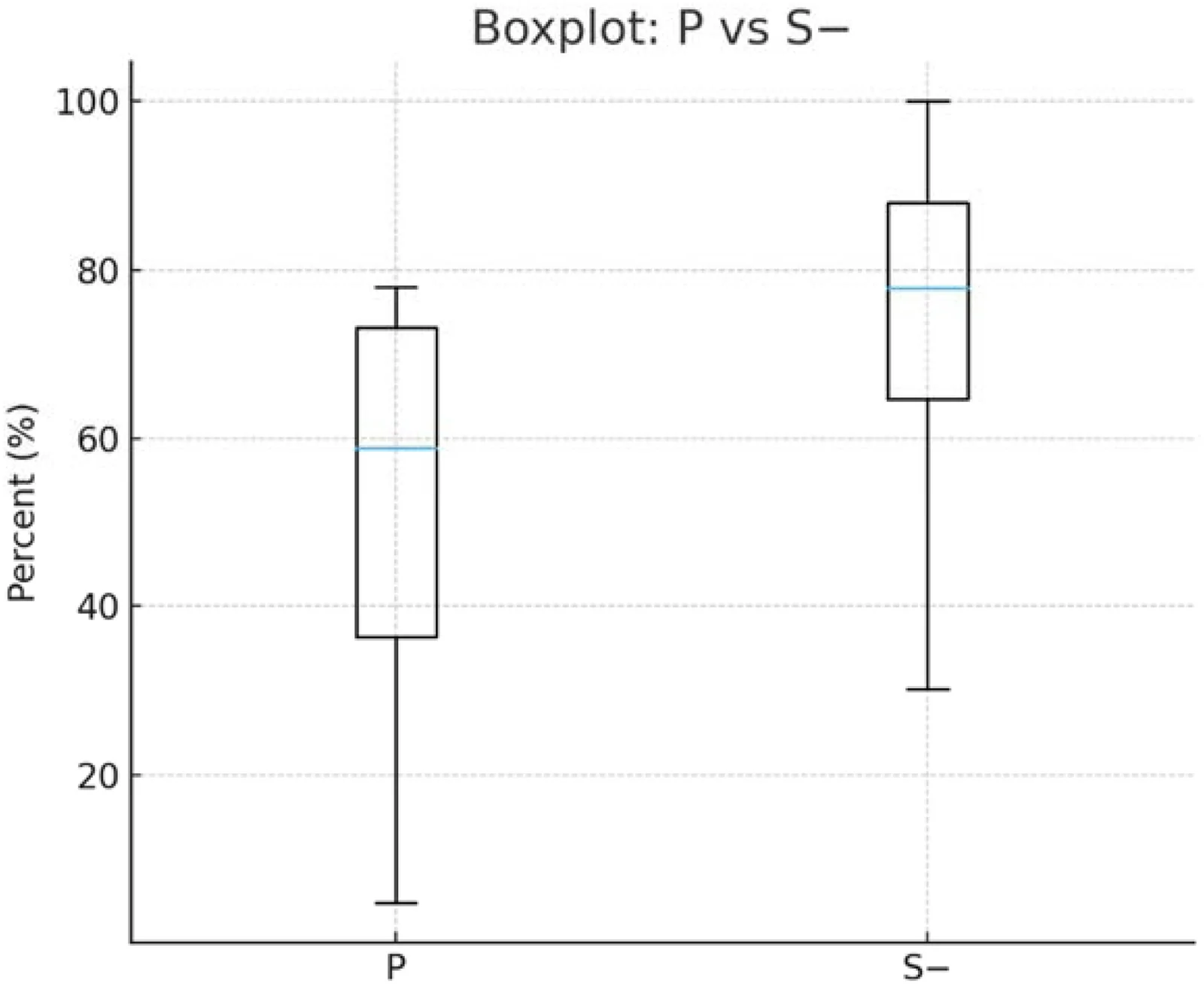
Boxplot showing the percentage of correct answers in the sentence test for strategies P and S.

## Discussion

The results of the present study strongly corroborate previous findings.^3^, ^4^, ^5^ In the present study, a statistically significant difference was observed between the two strategies, with superior performance for S (p < 0.001), with a high effect size (Hedges’ *g* = 1.38). The literature reports an average difference in sentence recognition between sequential and paired strategies of around 12%.^3^, ^4^, ^5^ In the study by Holcomb et al. (2021)^3^ and in that by Reynolds & Gifford,^5^ both using the AzBio test in noise, the mean difference between the strategies was approximately 12%, and most participants used Naída CI line processors (Q70 or Q90). In the German study by Buechner et al. (2005),^4^ which evaluated sentences in noise, the average difference between sequential and paired stimulation was similar, also in the range of approximately 12%, although the article does not specify the processor model used. Consistently, all studies showed more robust differences in tests performed in noise, a condition in which the impact of paired stimulation becomes more evident.

In the present study, the average difference between the strategies was 20.36%, reinforcing the clinical relevance of sequential stimulation. Most of our participants performed the assessment in silence, according to their initial performance (not exceeding 80%). In cases where performance exceeded 80%, following the protocol adopted by the service, the assessment was conducted in the presence of competitive noise, as this is a more challenging condition and more representative of everyday communication demands. As already noted by Buechner, many individuals achieve maximum performance (100%) in speech recognition tasks in silence, which limits the ability to detect differences between strategies in this context.^4^ Among our participants, only one showed ceiling performance in silence; for this case, we used the test in noise to enable comparison between strategies. Another individual performed the test in its slowed speed version, due to difficulty with the test at typical speed. It is important to note that of the 11 ears analyzed, 5 used Marvel line processors (M30/M90) and 6 used Quest line processors (Q70/Q90). This difference in technology is unlikely to have influenced the analysis, as each participant was compared with their own performance across strategies. Moreover, the underlying principle of the sequential strategy is similar across both technologies. Regardless of the device used, all participants showed better performance with the sequential strategy.

The study by Holcomb et al. was retrospective and, therefore, did not control the length of time each strategy was used, resulting in great variability among participants, who had anywhere from a few months to many years of experience with each condition.^3^ In the study by Reynolds and Gifford, each participant used six different strategies for two weeks, alternating them every two hours to minimize any adaptation effect.^5^ In the study by Buechner et al., each strategy was used for one month before speech recognition was assessed.^4^ In the present study, the tests were performed when the user arrived at the consultation with their usual map, as routinely occurs in clinical practice. After changing the strategy, the participant could be tested at the same time or after one month, depending on their availability, which is a limitation of the study related to the variability in the time of use of the new programming before reassessment. The limitations of this study are also related to the heterogeneity of the sample, both in terms of hearing performance and the technology in use, covering Quest and Marvel line processors. In addition, tests were customized according to each individual's performance level, which resulted in variations in the assessment conditions: most ears were tested at typical speed in silence, one was assessed at a signal-to-noise ratio of +5 dB, and another was assessed with the slowed speed version of the test. Although this heterogeneity can be seen as a methodological limitation, it also reflects the clinical reality, in which users have different performance levels and use different technologies. It is important to note that these factors were substantially controlled by the paired design of the study. By comparing each participant with themselves in the two strategies, the effects of interindividual variables such as age, length of implant use, hearing experience, or processor model are minimized. Thus, even given the diversity of the sample and the particularities of the tests used, the design adopted allowed for more accurate isolation of the impact of the change in strategy, preserving the clinical validity of the findings. Therefore, despite its natural limitations, the study remains highly relevant in practice, especially as it reflects real conditions of care and adaptation in clinical routine. Nevertheless, the small sample size is an important limitation, as it may influence the stability of statistical estimates. This aspect is evidenced by the relatively wide confidence interval observed for the effect size (Hedges’ *g*), indicating less precision in the actual magnitude of the effect, although the interval does not include the value zero, which reinforces the presence of a consistent effect. Thus, there is a need to replicate the results in larger samples in order to confirm the robustness of the findings and refine the estimate of the observed effect.

Furthermore, this is the first study conducted in Brazilian Portuguese ‒ and, to our knowledge, the first in a Latin language to directly compare paired and sequential strategies. It is possible that the degree of difference observed in speech recognition is influenced by linguistic characteristics of Portuguese, such as greater syllabic complexity and phonetic density when compared to English and German, reflecting even more striking differences between the strategies. Thus, this particularity highlights the unprecedented clinical relevance of the study, as it provides evidence directly applicable to the Brazilian population. In the current generation of processors available from the manufacturer (Marvel CI), strategy S is proposed as the standard stimulation. However, for users of previous technologies, it is recommended that audiologists check whether there are potential CI users who could benefit from switching from paired to sequential stimulation.

## Conclusion

This is the first study in Brazilian Portuguese ‒ and possibly the first in a Latin language ‒ to compare paired and sequential strategies in cochlear implant users. The results clearly show that the sequential strategy offers superior performance. Given this and considering that the brand’s standard strategy was recently updated with Marvel technology, it is recommended that audiologists systematically review their patients' programming to identify those who may benefit from migrating to the sequential strategy.

## ORCID ID

Aline Faria de Sousa: 0000-0002-7186-2471

Aline Santos: 0000-0002-2286-1405

Lucas Bevilacqua Alves da Costa: 0000-0003-3054-399X

Orozimbo Alves Costa: 0000-0002-4781-6096

Ana Claudia Martinho de Carvalho: 0000-0002-1767-0866

## Funding source

This study received no external funding.

*Institutional affiliation of the Institutional Review Board or Animal Use Committee that provided consent for the research: Research ethics Committee of the Prevent Senior Hospital Protocol or application number and Principal Investigator name submitted to the Institutional Review Board or Animal Use Committee for review of your research: Protocol number: nº 7.451.722. Principal Investigator: Lucas Bevilacqua Alves da Costa*.

## Data availability

The authors declare that all data are available in repository.

## Declaration of competing interest

Ana Claudia Martinho de Carvalho is employed by Advanced Bionics LLC. The other authors declare no conflicts of interest. Advanced Bionics had no role in the study design, data collection, data analysis, manuscript preparation, or decision to publish.
